# Evaluating patient experience to improve care in a specialist antenatal clinic for pregnancy after loss

**DOI:** 10.1186/s12884-023-06217-w

**Published:** 2024-01-10

**Authors:** Kajal K Tamber, Rebecca Barron, Emma Tomlinson, Alexander EP Heazell

**Affiliations:** 1 Maternal and Fetal Health Research Centre, University of Manchester, Manchester, UK; 2 Saint Mary’s Hospital, Manchester University NHS Foundation Trust, Manchester, UK

**Keywords:** Stillbirth, Antenatal care, Pregnancy, Rainbow clinic, Experience, Questionnaire

## Abstract

In the United Kingdom, roughly 1 in 250 babies are stillborn each year. Most women who experience stillbirth become pregnant again – 80% within a year of loss. Presently, obstetric-led care is recommended; though there is a growing body of evidence to support provision of specialist services. The Rainbow Clinic is a specialist antenatal service providing care for pregnancies after loss incorporating clinical and psychological care. This study aimed to assess patient experience at the Rainbow Clinic and identify areas for clinical improvement. A 13-item questionnaire was distributed to pregnant women who attended the Rainbow Clinics at the Oxford Road and Wythenshawe sites of Saint Mary’s Hospital, Manchester, UK between July 2016 and June 2021. Descriptive statistics and unpaired t-test were used for quantitative data and summative content analysis for qualitative data. Four-hundred and fifty-six women completed the questionnaire. The mean patient experience score per quarter was stable with an average of 21.1 (± 3.0) for the five years, with a maximum attainable score of 25. The COVID-19 pandemic had no effect on patient experience at the Rainbow Clinic (pre-pandemic vs. during-pandemic: mean 21.2 v 21.3; p = 0.75). Free-text responses demonstrated women felt positively about the antenatal care received. Identified areas for improvement included “more awareness of the [Rainbow] sticker” to ensure women with previous loss are identified; increased publicity of the Rainbow Clinic services; developing more clinics at different locations to improve accessibility; and continuing specialist input into intrapartum care. Specialist antenatal care provided by the Rainbow Clinic was rated as of a high standard. Potential future improvements include sticker alterations (or other mechanisms to identify women who have experienced a previous loss) and develop increased awareness of the clinic in other institutions.

**Supplementary Information:**

The online version contains supplementary material available at 10.1186/s12884-023-06217-w.

## Background

In the United Kingdom (UK), a stillbirth is defined as the birth of a baby at or after 24 weeks’ gestation showing no signs of life; in 2021, roughly 1 in 250 babies were stillborn [1]. The stillbirth rate in the UK was decreasing until 2021; there has been a 9% reduction since the implementation of NHS England’s Saving Babies’ Lives Care Bundle in 2015 [1, 2]. Nonetheless, a greater reduction is required to meet the Department of Health’s ambition to halve the stillbirth rate by 2025 (with a preliminary target of a 20% reduction by 2020) [3].

Women who have experienced a stillbirth are nearly five times more likely to have another stillborn baby compared to women with no such history (pooled odds ratio (OR) 4.83, 95% confidence interval (CI) 3.77 to 6.18; 16 studies, 3,412,079 births) [4]. This is pertinent as the majority of women who experience a stillbirth have a subsequent planned pregnancy, with 86% conceiving within 18 months [5]. Moreover, experiencing a stillbirth is a deeply distressing experience for mothers and their families, and can have significant psychological, social and financial implications [6, 7]. A recent meta-analysis including 19 studies reported women who have experienced a perinatal death have an increased risk of developing anxiety (d = 0.69, 95% CI: 0.41–0.97; p < 0.0001) and depression (d = 0.22, 95% CI: 0.15–0.30; p < 0.0001) in subsequent pregnancies, but no increase in stress levels (d = − 0.002, 95% CI: -0.06-0.06; p = 0.96) [7]. Women can have negative experiences in their future pregnancies due to a lack of continuity of care, needing to explain their loss numerous times, communication errors, and lack of psychosocial and emotional support [5, 6].

Specialist care is recommended in an attempt to mitigate some of the risks of pregnancy after a previous pregnancy loss [5]. The Royal College of Obstetricians and Gynaecologists (RCOG) recommend obstetric-led antenatal care with birth at a specialist maternity unit due to the increased risk of adverse outcomes [8]. Pregnant women who have experienced a stillbirth are more frequent users of antenatal healthcare services. The Norwegian Mother and Child Cohort Study [9] reported that women with a previous stillbirth had more antenatal visits than women with previous livebirth (mean 10.0 vs. 6.0, p < 0.001) or nulliparous women (mean 10.0 vs. 6.3, p < 0.001), as well as more ultrasound scans, more frequent unscheduled contact with their midwife and more hospital admissions. These data are consistent with a prior small-scale cohort study conducted in the United States of America (USA) [10] and an international survey [11].

In view of RCOG guidance, the risks associated with future pregnancies, and to incorporate psychosocial care, a specialist antenatal service caring for pregnancies after perinatal death (stillbirth or neonatal death), the Rainbow Clinic, was established at Saint Mary’s Hospital, Manchester in 2013. The Rainbow Clinic aims to improve the experience of antenatal care after loss and improve pregnancy outcomes whilst meeting women’s additional needs via additional clinical and psychological care; the care provided has been previously described [2]. In brief, women are offered consultant-led care with specialist midwifery support, regular ultrasound scans for fetal growth and umbilical and uterine artery Doppler ultrasound from 23 weeks’ gestation, a detailed birth plan including timing and mode of delivery, and access to specialist perinatal bereavement counselling. This service has since been expanded, initially in an adjacent maternity unit. It was deemed important to review patient experiences to determine whether the expanded service continued to achieve its objectives and whether any changes need to be made to improve service users’ outcomes. Therefore, this study aimed to quantify and describe the experiences of women attending the specialist antenatal clinics for pregnancies after perinatal death to determine whether high levels of patient satisfaction were consistently achieved and if not to identify areas for improvement in clinical care and patient experience.

## Methods

This was a retrospective study analysing patient experience at the Rainbow Clinic between July 2016 and June 2021 using a questionnaire-based approach at two sites in Saint Mary’s Managed Clinical Maternity Service in Central and South Manchester, UK. These maternity units serve a socially and ethnically-diverse population.

Pregnant women were eligible for inclusion if they attended the Rainbow Clinic during a pregnancy after perinatal death. No exclusion criteria were noted. Women were asked to complete a 13-item patient experience questionnaire (Additional File 1) at their final antenatal appointment at the Rainbow Clinic. This questionnaire was developed with input from three service-users and staff at the clinic who were independent from the evaluation team. The questionnaire was further tested in a small group of current service users and refined before being more widely used. The domains assessed included women’s emotions after the appointments, thoughts about number and duration of appointments, and involvement and planning of care. Twelve questions required participants to choose a pre-determined answer (quantitative analysis) followed by an additional free-text area for further explanation of their answers (qualitative analysis). Question 12 was excluded from any quantitative analysis. The final question was for qualitative analysis only.

### Quantitative analysis of questions 1–12

Quantitative data were assigned a score from − 2 to 2 (Table 1) and entered into a study database. Microsoft Excel was used for descriptive statistical analysis. An overall patient experience (PE) score was calculated for each individual using the sum of questions 1–11. The average PE score for each quarter of a year were calculated, where Q1 was inclusive of January-March, Q2 for April-June, Q3 for July-September and Q4 for October-December. Data were analysed using descriptive statistics and unpaired t-test. Data were presented using mean (± standard deviation (SD)) and/or using raw answer counts where appropriate. Run charts were used to determine whether there was a change in PE score over time.

**Table 1:** Coded numerical values for questions 1–11

Question(s)	Score		
	Explanation	Maximum rating	Minimum rating
1	1 point added for each positive emotion and 1 point deducted for each negative emotion	6	-6
2	Score of 1 for “appropriate number of appointments”. Score of -1 for “too few” or “too many”	n/a	n/a
3–11	Use a five-point Likert scale ranging from strongly agree (2) to strongly disagree (-2)	2	-2

### Qualitative analysis

Handwritten free-text responses from the paper patient experience questionnaires were transcribed in Microsoft Excel. Summative content analysis was utilised to analyse the final question, as outlined in Hsieh & Shannon [12]. First, the text was read and re-read several times. Then the text was closely analysed for surface and underlying meaning. Codes were identified independently by KT and RB. KT and RB discussed the codes with AH to validate and agree a consensus for coding. The content meaning of sentences or paragraphs within the free text responses transcribed were labelled with one of *n* codes. The number of meaning units or statements identified with a code was *n* in total. Code frequency was tallied and finally codes were categorised according to “positive code”, “negative code” or “code suggesting improvement”.

## Results

Four-hundred-and-fifty-six women completed the questionnaire; qualitative data were analysed from 357 questionnaires.

### Quantitative analysis

#### Overall patient experience

**Fig 1:**
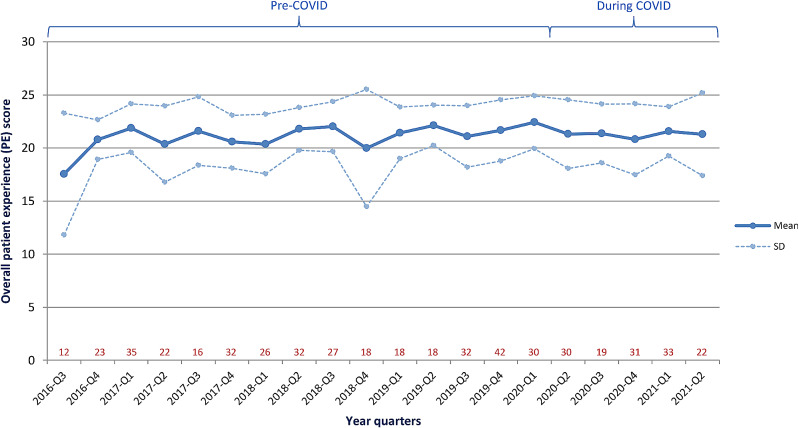
Impact of time on the overall patient experience score in women attending the Rainbow Clinic. The number of completed questionnaires for each quarter year is written in red. The maximum achievable score is 25. Mean and standard deviation (SD) for each quarter year is shown

Over the past five years, the mean PE score has been stable with an average of 21.1 (± 3.0) (Fig. 1). On two occasions, Q3 of 2016 and Q4 of 2018, the mean PE was less than 20.0 with an increased variation in responses. The COVID-19 pandemic, declared a UK emergency in March 2020, had no effect on patient experience at the Rainbow Clinic (pre-pandemic vs. during-pandemic: mean 21.2 vs. 21.3; p = 0.75).

#### Individual questions

Breakdown of the mean score for each question over the 5-year period is demonstrated in Table 2, all questions had a positive mean value with small disparities in the standard deviation. Exceptions to this were question 1 which had a unique scoring system compared to the other questions, and question 10 which demonstrated a lower mean with greater deviation from the mean. 97.3% of respondents believed they had an appropriate number of appointments at the Rainbow Clinic (question 2), whilst the remainder (2.7%) believed there was either an excess or lack of appointments.

**Table 2:** Mean score and standard deviation (SD) for questions 1, 3–11

Question	Domain assessed	Mean score	SD
1	Emotion after appointments	3.55	± 1.69
3	Adequate duration of appointments	1.81	± 0.41
4	Understanding & sympathetic staff	1.91	± 0.33
5	Concerns taken seriously	1.89	± 0.32
6	Feeling cared for	1.93	± 0.27
7	Care plan explained	1.90	± 0.33
8	Active role in care	1.88	± 0.37
9	Feeling listened to	1.89	± 0.37
10	Sticker prevented mistakes	1.51	± 0.79
11	Recommending clinic to others	1.95	± 0.23

Further analysis of individual questions revealed that only 66.4% of respondents strongly believed that the use of a ‘Rainbow sticker’ on their notes (to identify they had a prior loss) helped to prevent staff from making mistakes (question 10). This differs from the remainder of questions where between 81.0 and 95.1% of participants strongly agreed with the statements provided in the questionnaire.

### Qualitative analysis

**Fig 2:**
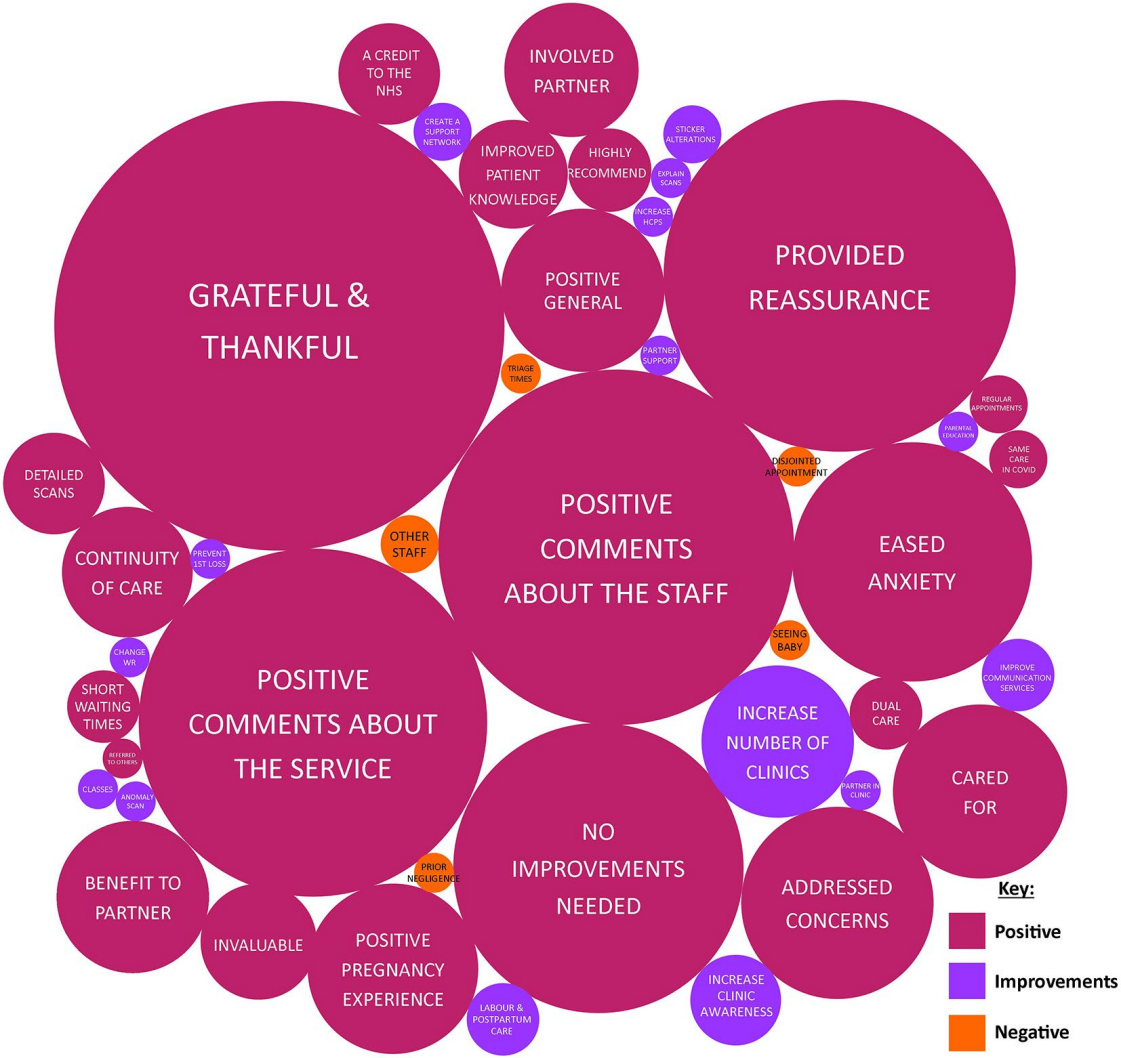
A bubble diagram of codes from free-text responses of the last question of the questionnaire. In total, there were 622 codes from patient responses. The area of each circle corresponds to the frequency of the code. Colours: pink = positive codes; purple = areas which participants identified for improvement; orange = negative codes

Using summative content analysis, 622 individual codes were derived from the 357 free-text responses; 92.0% of codes were categorised as positive (Fig. 2).

## Positive responses

### Gratitude to the staff and service

Women utilised the questionnaire to report gratitude to the Rainbow Clinic staff, specifically for the care provided throughout their pregnancy; this was accompanied with a variety of positive remarks. On numerous occasions, the women would specifically name members of staff who they wanted to give thanks to. Women also reported feeling supported by the staff, having all their concerns addressed at each appointment and feeling reassured that they were being provided with specialist care.

*We are extremely grateful for the Rainbow Clinic. They have looked after us so much and we have felt so safe and reassured.*

### Inclusion of partner & family in care

Whilst standard antenatal care has been primarily designed for pregnant women, responses indicated the service provided at the Rainbow Clinic was also beneficial to women’s partners and families - partners who attended felt included and that they were affected positively by attending Rainbow Clinic with their pregnant partner. This holistic approach eased anxieties surrounding pregnancy for -those who attended clinic. Furthermore, the staff can suggest resources and support groups available for the partners of pregnant women attending.

*I couldn’t thank [the staff] enough for everything they have done not only for my peace of mind but my partner’s and all my family involved.*

### Specific service benefits

Women reported several benefits of this specialised antenatal service on their experience of pregnancy. Firstly, the continuity of care enabled women to build relationships with healthcare professionals who understood and remembered their previous loss, this therefore removed the need for women to repeat the story of their previous loss(es). Women valued the use of their previous child’s name in conversations.

*Some things we found particularly helpful were: everyone knowing our history, not having to explain it to anyone; the use of our son’s name at our appointments - so important.* 

Secondly, the frequency of appointments received positive feedback as women felt they were able to adjust the timing of their next appointment as required, some women attended appointments as regularly as every fortnight.

*They always provided with the option of how soon I would like to re-attend. Rainbow Clinic - this was helpful to me.*

The access to detailed ultrasound scans at each appointment provided reassurance to women, in addition to providing improvement in patient knowledge regarding the health of their baby.

*Dr X explained everything on the scan and then after the scan I asked a lot of questions about how to monitor things very carefully towards the end and I felt she really improved my knowledge.*

Women also appreciated that they were not rushed during appointments, and generally felt very fortunate that the Rainbow Clinic existed to support them. A handful of women travelled from across the UK to attend. In these cases, the Rainbow Clinic provided collaborative care alongside women’s local maternity unit, suggesting care plans personalised to individual needs and circumstances.

### Recommendation

Women felt very positively about the impact the Rainbow Clinic had on their pregnancy and highly recommend it to others who have had a previous perinatal loss. One woman also reported this was her second pregnancy with the Rainbow Clinic.

*Highly recommend this clinic to anyone who has experienced the loss of a baby.*

### Reponses suggesting improvement

#### Increase services and public awareness

There were numerous responses requesting expansion of services to more hospitals and to different locations in the UK so women did not have to travel such distance to access this specialist service.

*Rolling this clinic out to the rest of the UK would allow all parents to experience the extra support needed.*

Alongside the increase in services, women also reported that the Rainbow Clinic required increased publicity and awareness. This will ensure that more pregnant women with a previous loss can access the clinic and healthcare professionals are aware of referral pathways – specifically general practitioners, community midwives and consultants at hospitals with no specialist service.

*Relatively few staff in my local hospital knew about the clinic including my consultant, so maybe some more publicity and awareness raising would be helpful about how the clinic exists and what it offers.*

#### Support services

The Rainbow Clinic offers support by providing a specialist telephone line to ring during working hours which women find helpful. However, suggested extensions of support services include Rainbow support groups to incorporate the experiences women and their partners might have when caring for a baby after experiencing a stillbirth in prior pregnancies. Women reported they felt reluctant to attend typical antenatal classes as their experience of pregnancy is different from those who haven’t experienced a loss. In addition, this extends to developing and providing “*more support for partners*” of pregnant women who have also experienced the loss of their baby.

*Perhaps a support group for other expectant parents so they can share feelings/experiences, so you don’t feel your reluctance/lack of say in pregnancy is a normal feeling.*

#### Rainbow sticker

Some women suggested improvements to the Rainbow Sticker attached to the front of antenatal paper notes to alert staff of a woman’s previous loss. An *“’online’ version […] for hospitals who used online notes”* would ensure that the Rainbow sticker could be used more widespread, as well as increasing awareness of the sticker on both a local and national scale to ensure healthcare professionals are aware of a woman’s previous loss.

*It would be great if across all antenatal/maternity there could be some sort of marker/sensitive alert on notes/systems to make sure families aren’t triggered by having to explain their story/anxieties to non-Rainbow staff.*

#### Negative responses

There were few negatives reported by women which included less considerate remarks made in other areas of the hospital as well as long waiting times in triage areas when women contacted them with concerns; however, antenatal triage runs independently to the Rainbow Clinic as an emergency and out-of-hours service.

*Other areas of the hospital […] aren’t always as considerate.*

Some women expressed specific feelings towards aspects of their care, such as a singular appointment feeling disjointed, wanting ultrasound scans more frequently than fortnightly to monitor baby and ease anxiety, and preparation for the change in a stillborn baby’s appearance after birth.

## Discussion

### Study findings

This quality improvement study demonstrated that women attending a specialist antenatal clinic for pregnancy after stillbirth had largely positive experiences. There are no directly comparable studies of specialist pregnancy after loss clinics, but many aspects assessed in the questionnaire received better responses than previously reported values, in which the majority of respondents were attending non-specialist antenatal care [11] (Table 3); including the appointments having an adequate duration, feeling listened to, women’s concerns taken seriously and having an active role in their antenatal care.

**Table 3:** Questionnaire responses of this quality improvement study compared against those reported by Wojcieszek et al. [11]. Study responses were appropriately matched to the rainbow clinic questionnaire

	Percentage of women from UK & Ireland who chose “always” in Wojcieszek et al. (11)	Percentage of women who chose “strongly agree” in this study.
Adequate duration of appointments	45.4%	81.0%
Understanding & sympathetic staff	57.6%	91.3%
Concerns taken seriously	51.1%	89.5%
Feeling cared for	58.6%	93.0%
Care plan explained	43.5%	90.7%
Active role in care	48.3%	89.0%
Feeling listened to	49.0%	90.1%

The stability of the mean PE score over the past five years is reassuring; particularly as it appears patient experience at the Rainbow Clinic was not adversely affected by the COVID-19 pandemic, which saw numerous services in other specialities cease and despite partners not being able to accompany pregnant women to their antenatal appointments. From a clinical point of view, it remains unclear why there were two drops in the mean PE score (Q3 of 2016 and Q4 of 2018) but this could correlate to a lower number of responses during these time periods; therefore, those with a less positive experience have a larger than usual effect on the overall score. In addition, it was shown that achieving a high and stable mean PE was quickly achieved by Q4 2016. Assessment of the rate of improvement in PE score would be an important goal for new Rainbow Clinics opening. Furthermore, with a mean PE of 21.1 (± 3.0) and a maximum achievable score of 25.0, it could be challenging to further positively increase women’s experiences although it is achievable, however first areas for improvement must be identified and addressed.

The qualitative aspect of the study found largely positive views; women who attended the Rainbow Clinic reported having positive experiences with the staff that communicate sensitively, were grateful for the increased monitoring provided and agreed that specialist care is delivered. Therefore, the quality of care provided by the Rainbow Clinic appears to be better than care provided in other areas of the UK, for a pregnancy after loss clinic [5]. It was encouraging that the Rainbow clinic was able to ease anxiety and provide reassurance to expectant women in their pregnancy after loss – one of the aims of the clinic. A meta-synthesis utilising 14 qualitative studies, to understand parent’s experiences of antenatal care after stillbirth, reported that women continue to experience profound ongoing grief and anxiety during pregnancy due to the loss of their previous baby, and noticed increased levels of anxiety when approaching the gestational age of their stillborn baby [13]. This is recognised by the staff at the Rainbow Clinic hence a planned appointment around the gestational age of their stillborn baby is offered to provide additional relief and psychological support. Pregnant women also reported that the Rainbow Clinic included partners and their family members, thereby providing an emotional benefit to partners. This is pertinent as a previous meta-analysis has reported that male-partners are at a greater risk of anxiety compared to their pregnant partners during pregnancy after loss [7].

The main negative finding of the study was related to the effectiveness of the Rainbow sticker to identify pregnant women with a previous stillbirth; ideally this sticker would have enabled the use of more sensitive language and prevention of difficult conversations. The majority of women (86.7%) agreed that the sticker prevented staff from making mistakes, similar to a previous finding from Heazell et al. [2]. However, it is important to acknowledge those who did not find the sticker useful; the main reason for this was due to healthcare professionals being unaware of the importance of the sticker, whether this was in other departments or in a different hospital. A simple solution to this is to educate healthcare professionals about the Rainbow Clinic sticker’s clinical importance and relevance in antenatal care; one means to deliver this would be an online learning package.

Pregnant women who have experienced loss often feel excluded from standard antenatal classes as they believe the class are not appropriate for them based on their background and often they do not want to discuss this with fellow expectant mothers [2]. The free-text questionnaire responses showed those attending the Rainbow Clinic wanted specialist antenatal classes for themselves and their partners; a similar response was also demonstrated by Mills et al. [5]. Attending tailored support programmes has been shown to have significant benefits as it enables open discussion about grief and the worries parents experience through the current pregnancy [13]. The use of peer support programmes is also recommended in the International Consensus Statement on care in pregnancy after loss [14].

### Limitations

There were several limitations of this quality improvement study. Firstly, due to time constraints and the volume of text provided by respondents, more sophisticated methods of qualitative analysis were unable to be employed (e.g. thematic analysis) [15]. Secondly, patient experience was not analysed alongside participant demographics to determine whether certain groups of women had a different experiences and care could be more suitably tailored for them. This occurred as this was an anonymous informal study with no mandatory questions, hence the majority of women didn’t complete the demographics section of the questionnaire and unfortunately, due to clinical data accessibility this information was not able to be retrieved. It would have been interesting to analyse the demographical relationship with patient experience, particularly as pregnant women from Black and Asian ethnic groups have a greater risk of stillbirth, and it would help understand whether the Rainbow Clinic is meeting the needs of all women during pregnancy after loss. Similarly, questionnaires were only provided in English, hence women who are unable to read and write English were not able to participate. Subsequent studies performed at the Rainbow Clinic have included access to interpreters to boost participation from non-English speakers. Data from the two centres (St Mary’s Hospital and Wythenshawe Hospital) were pooled together due to large disparities in the number of participants from each centre, ideally these would have been analysed separately and compared using formal statistical analysis to determine centre-specific improvements. Lastly, the possibility of responder bias should be considered, such that women who held negative views or experiences of the service may have chosen not to participate.

## Conclusions

The Rainbow Clinic provides specialist antenatal care to women with a previous loss of a high standard and was viewed favourably by women attending. Although, the vast majority of responses received were positive, every service can be improved to achieve clinical excellence, and any negative comments and those suggesting improvements must be acted upon. The next steps for the Rainbow Clinic include alterations to the Rainbow sticker attached to women’s notes and the development of an e-version of this to add to electronic notes, as well as the development of more Rainbow Clinics throughout the country. As the clinics are rolled out, future studies should compare patient experience before and after establishment of specialist clinical services. Furthermore, the outcomes of women attending this service could be compared to those from other specialist services e.g. diabetes or hypertension clinics or mainstream antenatal services. In the future, an additional aim would be the development of specialist antenatal classes for pregnancy after loss in replacement of standard antenatal classes, to enable interaction and discussion with other expectant parents with a similar history.

### Electronic supplementary material

Below is the link to the electronic supplementary material.

Supplementary Material 1

## Data Availability

The datasets used and/or analysed during the current study are available from the corresponding author on reasonable request.

## Declarations

### Authors’ information

KT is an Academic Foundation Doctor at Manchester University NHS Foundation Trust. She had a Masters of Research in Pregnancy and Reproduction from the University of Manchester and has a keen research interest in Women’s Health and Obstetrics.

AH is a Professor of Obstetrics and a Consultant Obstetrician. He is the Director of the Tommy’s Stillbirth Research Centre and he established Rainbow Clinic at Saint Mary’s Hospital in Manchester in 2013 and is the Chief Investigator on the Tommy’s National Rainbow Clinic Study (IRAS 249991).

## Abbreviations

CI: Confidence interval
HRA: Health research authority
OR: Odds ratio
PE: Patient experience (score)
RCOG: Royal college of obstetricians and gynaecologists
SD: Standard deviation
UK: United Kingdom
USA: United States of America
