# Supplementary material for: Evaluating patient experience to improve care in a specialist antenatal clinic for pregnancy after loss
Source: BMC Pregnancy Childbirth. 2024 Jan 10;24:51. doi: 10.1186/s12884-023-06217-w (PMC10777522; doi:10.1186/s12884-023-06217-w)
Supplement: Supplementary file 1 — Supplementary Material 1 [file 12884_2023_6217_MOESM1_ESM.pdf]

[Hospital Name]

Maternity Service - Rainbow Clinic

Patient Questionnaire

We are keen to hear your thoughts about your recent appointments in the Rainbow Clinic. This information will help us to improve this experience for other patients where we can. This survey should only take 4-5 minutes to complete.

Please circle the words that best describe your feelings or write your own words in the spaces provided:

Yes No

I think that...

1. Following your appointments in the Rainbow Clinic, did you usually feel: (Please circle all that apply)

Happy	Supported	Safe	Good	Comfortable	Worried
Lonely	Sad	Anxious	Reassured	On Edge	Other: (Please specify)

Why did you feel like this?

2. Thinking about your appointments, were there:

Too few	An appropriate number	Too many

Why did you feel like this?

3. I felt I had enough time in my appointment.

Strongly agree	Agree	Neither agree nor disagree	Disagree	Strongly disagree
○	○	○	○	○

If not, why not?

4. I felt that the Rainbow clinic staff were understanding and sympathetic to my previous experience

Strongly agree	Agree	Neither agree nor disagree	Disagree	Strongly disagree
○	○	○	○	○

If not, why not?

5. At my appointments I felt that my concerns were taken seriously and tests/scans were arranged where needed.

Strongly agree	Agree	Neither agree nor disagree	Disagree	Strongly disagree
○	○	○	○	○

If not, why not?

6. During my pregnancy in Rainbow Clinic I felt cared for by experienced professionals:

Strongly agree	Agree	Neither agree nor disagree	Disagree	Strongly disagree
○	○	○	○	○

If not, why not?

7. During my pregnancy, the plan for my care was discussed and explained to me, including frequency of appointments, scans, tests and additional considerations (such as taking aspirin, or other medications specific to my situation).

Strongly agree	Agree	Neither agree nor disagree	Disagree	Strongly disagree
○	○	○	○	○

If not, why not

8. During my pregnancy I felt sufficiently involved in decisions about my care:

Strongly agree	Agree	Neither agree nor disagree	Disagree	Strongly disagree
○	○	○	○	○

If not, why not?

9. Regarding communication with the midwives and doctors in Rainbow Clinic, did you feel listened to?

Strongly agree	Agree	Neither agree nor disagree	Disagree	Strongly disagree
○	○	○	○	○

If not, why not

10. The Rainbow Clinic Sticker helped to prevent staff from making mistakes, such as not knowing that my baby died in the past.

Strongly agree	Agree	Neither agree nor disagree	Disagree	Strongly disagree
○	○	○	○	○

11. I would recommend the Rainbow Clinic to another family who had experienced the death of a baby.

Strongly	Agree	Neither agree	Disagree	Strongly
○	○	○	○	○

If not, why not?

12. How did you find out about the Rainbow Clinic?

Referred by a GP	Referred by a midwife	Referred by a consultant
Support Group	Online	Other

Equality Monitoring Form

Central Manchester University Hospitals NHS Foundation Trust is committed to ensuring that everyone has equal access to **NHS services**. We would like your help to do this by answering a few questions about your background. The information you give us will be kept confidential and will be used to improve the quality of our service. Completion of this form is optional.

Thank you for your time.

Please tick ☑ the box that applies to you. If none of the boxes apply please write your answer in the space provided.

**1. ETHNIC ORIGIN**	**a) White**	
	British	☐
	Irish	☐
	Other (please specify):	
	**b) Mixed**	
	White and Black Caribbean	☐
	White and Black African	☐
	White and Asian	☐
	Other (please specify):	
	**c) Asian or Asian British**	
	Indian	☐
	Pakistani	☐
	Bangladeshi	☐
	Other (please specify):	
	**d) Black or Black British**	
	Caribbean	☐
	African	☐
	Other (please specify):	
	**e) Other Ethnic Groups**	
	Chinese	☐
	Other (please specify):	
	**f) Prefer not to say**	☐

**2. DISABILITY**	Do you consider yourself to have a disability?<br>Yes ☐ No ☐
**3. RELIGION**	Buddhist ☐<br>Christian ☐<br>Hindu ☐<br>Jewish ☐<br>Muslim ☐<br>Sikh ☐<br>Other (please specify): …………………………<br>No religion ☐<br><br>Prefer not to say ☐
**4. FIRST LANGUAGE**	Is English your first language?<br>Yes ☐ No ☐<br><br>If No, My first language is: ………………………
**5. GENDER**	Male ☐<br>Female ☐<br>Prefer not to say ☐
**6. AGE**	…………………………………………………………
**7. SEXUAL ORIENTATION**	Heterosexual ☐<br>Lesbian ☐<br>Bi-sexual ☐<br>Prefer not to say ☐

13. Your comments or suggestions for improving our service would be very welcome.

Thank you for taking the time to complete this questionnaire.
When you have finished, please hand this back to a member of staff in the department.

[INSERT CONTACT DETAILS]

[NAME AND EMAIL]

Produced Sept. 2016
